# Hyperbaric Oxygen Sensitizes Anoxic Pseudomonas aeruginosa Biofilm to Ciprofloxacin

**DOI:** 10.1128/AAC.01024-17

**Published:** 2017-10-24

**Authors:** Mette Kolpen, Christian J. Lerche, Kasper N. Kragh, Thomas Sams, Klaus Koren, Anna S. Jensen, Laura Line, Thomas Bjarnsholt, Oana Ciofu, Claus Moser, Michael Kühl, Niels Høiby, Peter Ø. Jensen

**Affiliations:** a Department of Clinical Microbiology, Rigshospitalet, Copenhagen, Denmark; b Department of Immunology and Microbiology, Costerton Biofilm Center, Faculty of Health and Medical Sciences, University of Copenhagen, Copenhagen, Denmark; c Biomedical Engineering, Department of Electrical Engineering, Technical University of Denmark, Lyngby, Denmark; d Marine Biological Section, Department of Biology, University of Copenhagen, Helsingør, Denmark; e Climate Change Cluster, University of Technology Sydney, Sydney, Australia

**Keywords:** biofilms, ciprofloxacin, hyperbaric oxygen, oxygen radicals, Pseudomonas aeruginosa

## Abstract

Chronic Pseudomonas aeruginosa lung infection is characterized by the presence of endobronchial antibiotic-tolerant biofilm, which is subject to strong oxygen (O_2_) depletion due to the activity of surrounding polymorphonuclear leukocytes. The exact mechanisms affecting the antibiotic susceptibility of biofilms remain unclear, but accumulating evidence suggests that the efficacy of several bactericidal antibiotics is enhanced by stimulation of aerobic respiration of pathogens, while lack of O_2_ increases their tolerance. In fact, the bactericidal effect of several antibiotics depends on active aerobic metabolism activity and the endogenous formation of reactive O_2_ radicals (ROS). In this study, we aimed to apply hyperbaric oxygen treatment (HBOT) to sensitize anoxic P. aeruginosa agarose biofilms established to mimic situations with intense O_2_ consumption by the host response in the cystic fibrosis (CF) lung. Application of HBOT resulted in enhanced bactericidal activity of ciprofloxacin at clinically relevant durations and was accompanied by indications of restored aerobic respiration, involvement of endogenous lethal oxidative stress, and increased bacterial growth. The findings highlight that oxygenation by HBOT improves the bactericidal activity of ciprofloxacin on P. aeruginosa biofilm and suggest that bacterial biofilms are sensitized to antibiotics by supplying hyperbaric O_2_.

## INTRODUCTION

Chronic pulmonary infection with Pseudomonas aeruginosa in cystic fibrosis (CF) patients is the first biofilm infection described in humans (1). In CF patients, chronic lung infection with P. aeruginosa constitutes the major cause of increased morbidity and mortality (2). Therefore, the dramatically increased tolerance of P. aeruginosa biofilms to antibiotics is a critical challenge for improving antibiotic treatment of chronic lung infections in CF patients (3). Increased tolerance of P. aeruginosa biofilms to antibiotics is multifactorial (4) and may to some extent depend on restriction of molecular oxygen (O_2_) (5, 6), which is distributed at low levels, reaching anoxia in parts of the endobronchial secretions of chronically infected CF patients (7–9). Since O_2_ is a prerequisite for aerobic respiration, shortage of O_2_ may decelerate aerobic respiration, leading to increased tolerance to several antibiotics (10–12). This enhanced tolerance possibly relies on decreased expression of antibiotic targets and antibiotic uptake (13) as well as reduced endogenous lethal oxidative stress in response to downstream events resulting from interaction between drugs and targets (11, 12). Accordingly, we have previously shown that reoxygenation of O_2_-depleted P. aeruginosa biofilms using hyperbaric oxygen treatment (HBOT) increases the susceptibility to ciprofloxacin. In that study the O_2_ was removed by bacterial aerobic respiration (14). However, this may be in contrast to the consumption of O_2_ in the endobronchial secretions of CF patients, in which the vast majority of O_2_ is consumed by the polymorphonuclear leukocytes (PMNs) for production of reactive O_2_ species (ROS) and nitric oxide (NO), whereas only a minute part of O_2_ is consumed by aerobic respiration (8, 15). In fact, ongoing anaerobic respiration and low *in vivo* growth rates of P. aeruginosa biofilms (16) and of several other bacterial pathogens (17–19) suggest limited bacterial aerobic respiration (20). Therefore, in order to mimic situations in CF lungs where intense O_2_ consumption by activated PMNs prevents engagement of bacterial aerobic respiration we have grown bacterial biofilm without O_2_ prior to antibiotic treatment and HBOT. Using this approach, we aimed to examine if absent aerobic respiration may be restored by HBOT for clinically relevant durations, leading to increased bactericidal effect of ciprofloxacin.

## RESULTS

### Effect of HBOT on P. aeruginosa biofilm during ciprofloxacin treatment.

**FIG 1:**
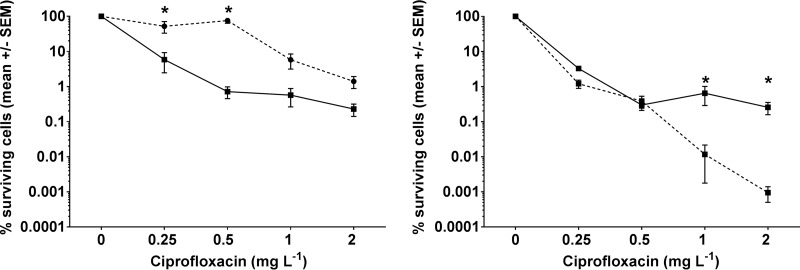
Effect of simultaneous hyperbaric oxygen treatment (HBOT) on ciprofloxacin (0.25 to 2 mg · liter^−1^) treatment of anaerobic Pseudomonas aeruginosa biofilms. (Left panel) Effect of anoxic (dotted line) and HBOT (solid line) conditions on % surviving cells on agarose-embedded PAO1 biofilms treated with ciprofloxacin (calculated as Δlog_10_ cell numbers) after treatment for 90 min. Bars indicate the mean ± standard error of the mean (*n* = 13 to 19). (Right panel) Effect of ciprofloxacin- and HBOT on 3-day-old agarose-embedded biofilms of PAO1 (solid line) and *ΔkatA* (dotted line) (calculated as Δlog_10_ cell numbers) after treatment for 90 min. Bars indicate the mean ± standard error of the mean (*n* = 11 to 14). Significant changes (*P* ≤ 0.05) by particular ciprofloxacin concentrations are indicated by asterisks (*). Statistical significance was evaluated by a two-way ANOVA test followed by Bonferroni's multiple comparison tests.

Significantly less PAO1 bacteria survived 90 min of treatment with ciprofloxacin when HBOT was applied (*P* < 0.0001, *n* = 13 to 19) (Fig. 1, left panel). The maximum enhancement of bacterial killing by HBOT exceeded 2 log units when supplemented with 0.5 mg · liter^−1^ of ciprofloxacin, indicating that P. aeruginosa biofilm exposed to HBOT can be treated with lower ciprofloxacin concentrations than controls.

It is striking that the potentiation of ciprofloxacin is stronger after 90 min of HBOT than for 2 h of HBOT as previously reported (14). However, the present model has been developed to better represent the *in vivo* microenvironment where P. aeruginosa is deprived of O_2_ due to intense O_2_ depletion by the surrounding PMNs creating anoxia (8). Furthermore, the depth of the agarose-embedded biofilm has been decreased in order for O_2_ to penetrate through large parts of the entire biofilm within 90 min.

In P. aeruginosa a major part of the detoxification of ROS is contributed by catalase enzymes encoded by the *katA* gene (21, 22). Accordingly, the increased susceptibility to antibiotics in mutants with defective *katA* expression, as well as the enhanced tolerance to antibiotics in mutants with overexpression of catalase, is recognized as direct evidence for a lethal effect of ROS generation during antibiotic treatment (12, 23, 24).

Therefore, we employed Δ*katA* biofilms to elucidate that ROS play a role in the increased lethality of ciprofloxacin during HBOT. We found significantly fewer Δ*katA* bacteria surviving 90 min of treatment with ciprofloxacin when HBOT was applied compared with PAO1 biofilms (*P* < 0.0024, *n* = 11 to 14), demonstrating a contribution of oxidative stress to decreased bacterial survival (Fig. 1, right panel). This indicates that HBOT enabled aerobic respiration, allowing ciprofloxacin to induce formation of lethal amounts of ROS (10). However, increased susceptibility of Δ*katA* was only seen for the higher concentrations of ciprofloxacin, suggesting that other antioxidative mechanisms protect against the ROS produced during treatment with small amounts of ciprofloxacin (10).

### HBOT expands the bactericidal zone of ciprofloxacin treatment in P. aeruginosa biofilm.

**FIG 2:**
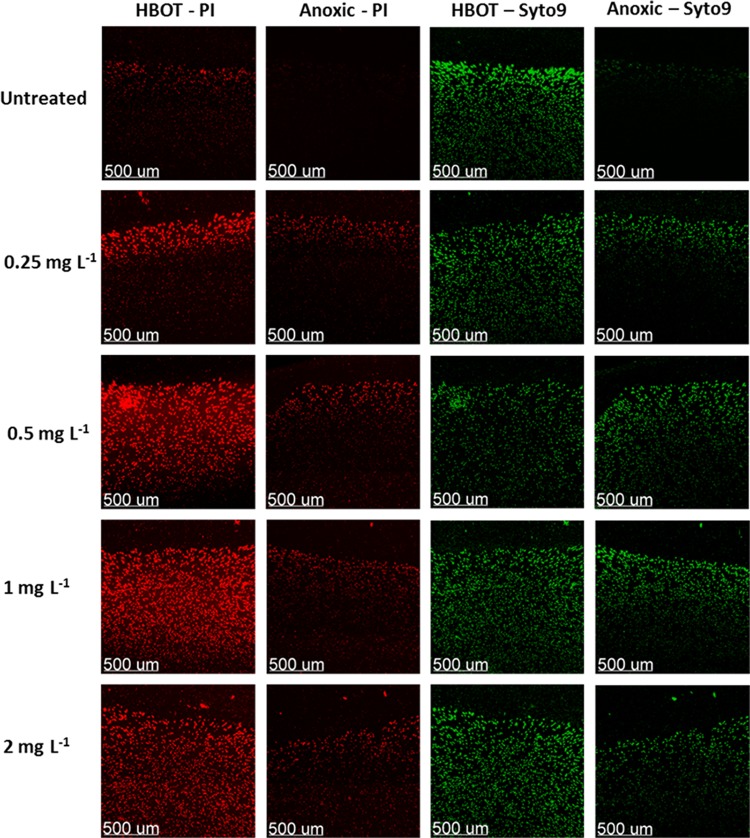
Lethality of ciprofloxacin-treated agarose-embedded Pseudomonas aeruginosa biofilms during anoxic or HBOT conditions. Visualization of representative 90-min ciprofloxacin and HBO-treated 3-day-old agarose-embedded biofilms of PAO1. Ciprofloxacin (0.25 to 2 mg liter^−1^) treatment in anoxic agarose-embedded biofilms of PAO1 and in HBOT agarose-embedded biofilms of PAO1. Samples were stained with Syto9 and propidium iodide (PI) and obtained using a 63 × 1.4 numerical aperture (NA) Zeiss objective on a Zeiss 710 CLSM. Red denotes bacterial membranes that are permeable to PI (dead bacteria); green bacteria are alive, since they have intact membranes that are not permeable to PI. The bar in the photograph represents 500 μm. (*n* = 1).

P. aeruginosa embedded in agarose that grows in discrete aggregates was detected by confocal microscopy (Fig. 2) (25). Variations in aggregate size may depend on whether initiation is from single or multiple cells. Aggregate diameter was significantly larger after 90 min of HBOT (100% O_2_, 280 kPa) than after anoxia (median diameter [range]: 37 μm (9 to 193 μm) versus 23 μm (7 to 66 μm); *P* < 0.0001, *n* = 139) estimated from live/dead staining of samples without ciprofloxacin treatment in the upper 100 μm of the agarose-embedded biofilm. Aggregate volume was 4.2-fold greater after 90 min of HBOT than after anoxia (median volume [μm^3^]: 27 versus 6.4, *n* = 139), indicative of 4.2-fold more bacterial cells and an additional 2 divisions compared to anoxic treatment. Furthermore, the propidium iodide (PI) experiments were intended to confirm the statistically significant difference found with CFU counting and to visualize the increased zone of bactericidal activity caused by HBOT during ciprofloxacin treatment.

### HBOT stimulates growth in P. aeruginosa biofilm.

**FIG 3:**
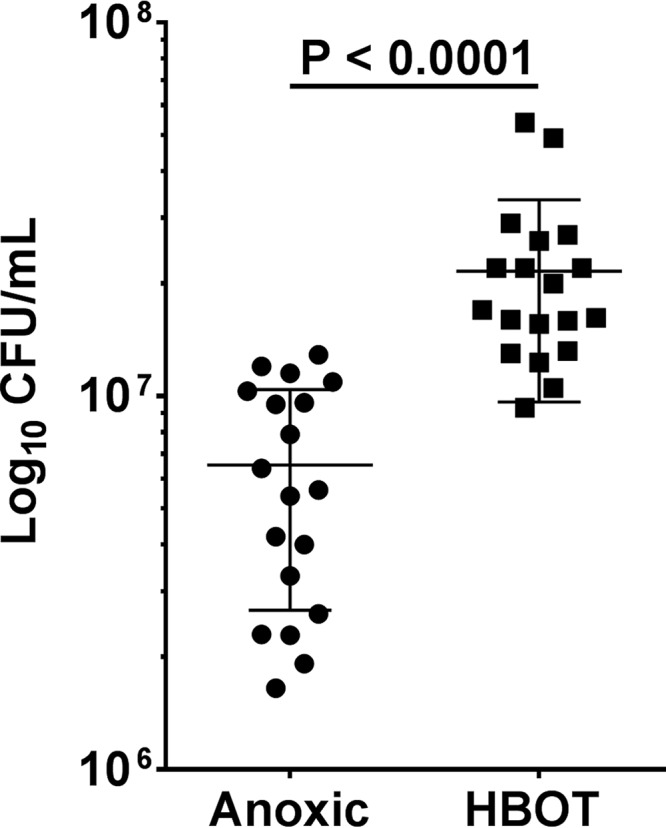
Hyperbaric oxygen treatment (HBOT) effect on bacterial growth in Pseudomonas aeruginosa biofilms. Effect of anoxic (circles) and HBOT (squares) conditions on bacterial growth (calculated as Δlog_10_ cell numbers) after treatment for 90 min on agarose-embedded PAO1 biofilms. Bars indicate the mean ± standard error of the mean (SEM) (*n* = 19). Statistical significance (*P* ≤ 0.05) was evaluated by the Student's *t* test.

Untreated PAO1 biofilms embedded in agarose were exposed to HBOT, with significantly increased bacterial growth demonstrated during the 90 min of incubation (*P* < 0.0001, *n* = 19). Compared with growth under anoxic conditions, HBOT increased the density of PAO1 biofilms without antibiotic treatment, indicating that aerobic respiration increases bacterial growth (Fig. 3). In fact, 90 min of HBOT increased bacterial growth by ½ log compared to anaerobic growth.

### Distribution of O_2_ in P. aeruginosa biofilm after HBOT.

**FIG 4:**
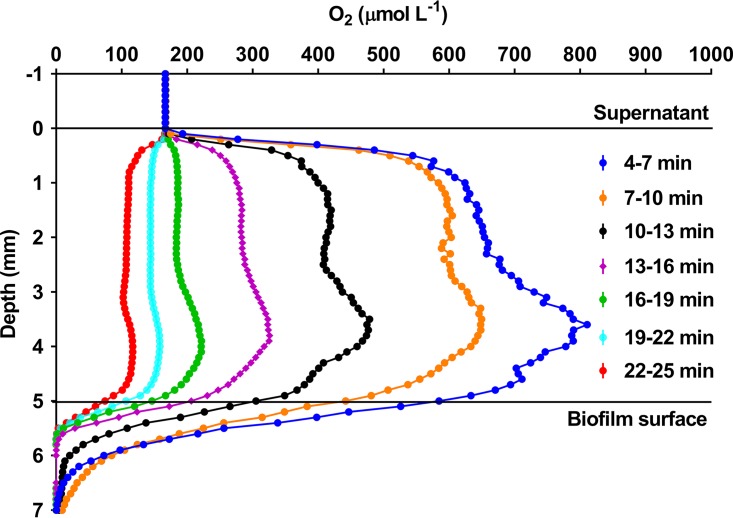
Optical microsensor measurement of the chemical gradient of O_2_ in ciprofloxacin-treated agarose-embedded Pseudomonas aeruginosa biofilm. Representative microprofiling of the spatiotemporal dynamics of O_2_ in an agarose-embedded PAO1 biofilm receiving HBOT for 90 min showing initial accumulation of O_2_ in the media above the biofilm surface and inside the biofilm, followed by depletion. The measurement of the O_2_ concentration profile was initiated 4 min after termination of HBOT with following profiling.

Vertical profiling of O_2_ concentration in the agarose-embedded biofilm immediately after termination of 90 min of HBOT demonstrated O_2_ concentrations exceeding 1,000 μmol · liter^−1^ in the media above the biofilm surface (Fig. 4). Serial profiling revealed both rapid depletion of O_2_ in the upper part of the biofilm and O_2_ diffusion from the supernatant to the normobaric atmosphere. However, within 20 min post HBOT, the zone of O_2_ depletion inside the biofilm was expanded and the O_2_ concentration of the supernatant decreased below atmospheric saturation, indicating that PAO1 was utilizing the available O_2_ for aerobic respiration until O_2_ depletion in the biofilm would necessitate conversion to anaerobic respiration (Fig. 4).

O_2_ diffusion through the agarose gel alone was detected at agarose concentrations from 0.125% to 2%. As expected (26), no significant concentration dependence or deviation from free diffusion was observed and accordingly the assumption was made that O_2_ diffusion is not hindered by agarose or water in the biofilm model (data not shown).

Ciprofloxacin efficacy is known to be linked to growth in view of the quinolone target's increased activity during DNA replication, both planktonically and in biofilms (27, 28). However, the inability to respire during aerobic respiration allows bacteria to arrest growth in a manner that increases tolerance. This study shows that addition of O_2_ sensitizes bacteria by stimulating growth in areas deprived of O_2_. It has been shown previously that quinolones also have a bactericidal effect on flow cell biofilms but that subpopulations remained tolerant to treatment. Similarly, our results on nonattached biofilm, reflecting a more accurate representation of chronic lung infection, show that the bactericidal effect of ciprofloxacin improved with HBOT.

## DISCUSSION

P. aeruginosa is clinically a very important respiratory pathogen that causes the most severe complication of chronic lung infection in CF patients (2). Throughout the chronic infection state, microbial biofilms form as cell aggregates and become trapped in the endobronchial mucus (29), with the host response creating chemical microenvironments favoring bacterial physiology associated with tolerance against multiple antibiotics (20). Therefore, new treatment strategies are required to overcome these resilient bacterial infections. HBOT has beneficial effects on the treatment of a number of infectious diseases, clinically, experimentally and *in vitro* (14, 20, 30), although whether these can be expanded to biofilm infections has not been extensively examined. The present study utilized a model in which anoxic P. aeruginosa was embedded in an agarose gel, trapping bacteria as aggregates throughout the gel in order to mimic biofilm infection *in vivo* (14, 30–32).

Few studies have shown that HBOT can be used as an adjuvant to ciprofloxacin treatment on P. aeruginosa (33, 34) and to our knowledge our recently published proof-of-concept study provided the first demonstration that HBOT can enhance the bactericidal activity of ciprofloxacin on biofilms (14). In the present study, it has been substantiated that bactericidal activity of ciprofloxacin is enhanced after only 90 min of HBOT, representing a typical time frame used clinically for HBOT (35, 36). The Undersea and Hyperbaric Medical Society recommends 90 to 120 min of HBOT per session (37). Prior to HBOT, bacterial growth supported by aerobic respiration in the biofilm model was prevented by O_2_ exclusion while addition of NO_3_^−^ enabled anaerobic respiration by denitrification (38, 39). The rapid decrease from hyperoxia to hypoxia demonstrated by serial measurements of O_2_ concentration profiles in the biofilm immediately after HBOT indicated engagement of aerobic bacterial respiration during HBOT, with this metabolic shift likely explaining the observation of faster growth of PAO1 under HBOT (40). Induction of increased metabolic activity by HBOT was further indicated by increased SYTO9 fluorescence intensity and bacterial aggregate size after HBOT, resembling colonies in metabolically active zones in similar biofilm models (25, 31).

Consequentially, activation of aerobic respiration by HBOT may contribute to the enhanced bactericidal activity of ciprofloxacin by accelerating bacterial growth, as the susceptibility to ciprofloxacin of P. aeruginosa biofilm is correlated to growth rate (41).

In addition to a growth-related enhancement of ciprofloxacin treatment during HBOT, it was speculated that HBOT-induced reoxygenation of the biofilm leads to accumulation of cytotoxic ROS in response to ciprofloxacin. Induction of endogenous production of cytotoxic ROS has been shown to contribute to the aerobic killing of planktonic bacteria by several major classes of antibiotics (11, 12, 42), including aerobic P. aeruginosa biofilms (43), although the significance of this has been challenged (11, 44, 45). However, increased susceptibility to antibiotics of mutants with deficient anti-oxidative defense is regarded as solid indication for a contribution of ROS to the bactericidal effect of antibiotics (23). Thus, the increased killing of the Δ*katA* mutant in our study supports that endogenous generation of ROS can contribute to an enhanced bactericidal effect of ciprofloxacin on biofilm during adjuvant HBOT. Growth of Δ*katA* was not impaired with HBOT in the absence of ciprofloxacin treatment compared to the wild type, indicating a lack of cytotoxic ROS generation by HBOT alone (data not shown).

Biofilm infections are notoriously difficult to eradicate with antimicrobial treatment, as higher concentrations of antibiotics are frequently required for killing of biofilms compared to planktonic bacteria, with these concentrations being difficult to match *in vivo* (46). Our finding of significantly increased bacterial killing during HBOT with only 2× MIC and 4× MIC of ciprofloxacin indicates that by using HBOT, P. aeruginosa biofilms can be effectively treated with lower ciprofloxacin levels that are attainable *in vivo*.

Although still controversial, there is an increasing acceptance of the advantages of HBOT, with a small number of studies focusing on the use of HBOT on biofilm infections, e.g., those associated with periodontal disease, osteomyelitis, and chronic wounds (47–49). The effect of HBOT on biofilm infections in the pulmonary system remain largely unknown, although some studies have demonstrated the beneficial effect of HBOT in patients with acute abscesses and in experimental pulmonary infection models with P. aeruginosa (50, 51). The feasibility of HBOT to sensitize infectious biofilm to antibiotics in patients is indicated by the fact of PAO1 being a clinical isolate from a burn wound (52, 53). In addition, we have recently demonstrated potentiation of tobramycin by HBOT on both *in vitro* and *in vivo* biofilms of clinical isolates of Staphylococcus aureus (54). However, a better understanding of the usefulness of HBOT in CF patients awaits further experiments with pathogens isolated longitudinally, as well as with isolates with known resistance, including highly resistant strains. The risk of development of barotrauma in the lungs, however, should raise concerns when applying HBOT to patients with severely damaged lung tissue.

In summary, the findings of this study point to a new treatment strategy for biofilm infections by providing HBOT as an adjuvant to ciprofloxacin treatment, where the increased availability of O_2_ leads to an increased susceptibility of P. aeruginosa biofilms to clinically relevant concentrations of antibiotic.

## MATERIALS AND METHODS

### Bacterial strains, media and antibiotics.

Wild-type P. aeruginosa strain PAO1 was obtained from the Pseudomonas Genetic Stock Center (http://www.pseudomonas.med.ecu.edu). Both the wild type and a catalase-A-negative PAO1 (*ΔkatA*) mutant (22) were tested for susceptibility to the bactericidal antibiotic ciprofloxacin (Bayer GmbH, Leverkusen, Germany). *katA* encodes the catalase enzyme responsible for the major part of detoxification of ROS in P. aeruginosa and accordingly the *ΔkatA* mutant was chosen to demonstrate ROS contribution to ciprofloxacin activity. The MIC of PAO1 was 0.125 mg · liter^−1^ as determined by Etest (bioMérieux, Ballerup, Denmark). Growth was in lysogeny broth (LB) (5 g · liter^−1^ yeast extract [Oxoid, Basingstoke, UK], 10 g · liter^−1^ tryptone [Oxoid], and 10 g · liter^−1^ NaCl [Merck, Rahway, NJ], pH 7.5), incubated overnight at 37°C and shaken at 150 rpm. For determination of bacterial CFU counts, solid lactose agar plates (“Blue plates” based on a modified Conradi-Drigalski medium containing 10 g · liter^−1^ detergent, 1 g · liter^−1^ Na_2_S_2_O_3_ · H_2_O, 0.1 g · liter^−1^ bromothymolblue, 9 g · liter^−1^ lactose, and 0.4 g · liter^−1^ glucose, pH 8.0; Statens Serum Institut, Copenhagen, Denmark) were used to select for Gram-negative bacteria. All plates were incubated overnight at 37°C.

### Anaerobic growth.

P. aeruginosa biofilms were grown and treated under anoxic conditions in an anaerobic growth chamber (Concept 400 Anaerobic Workstation, Ruskinn Technology Ltd., UK). The gas atmosphere consisted of N_2_/H_2_/CO_2_ (ratio, 80:10:10). Anoxia was confirmed with an optical O_2_ sensor (HQ40d Portable multi meter; HACH Company, CO, USA) placed in the growth chamber. To remove traces of O_2_, all media and chemical solutions applied for anaerobic work were equilibrated in the anaerobic chamber 3 days prior to experiment.

### Susceptibility testing of mature biofilms.

Survival curves were assayed to investigate the effect of HBOT on P. aeruginosa biofilms treated with ciprofloxacin during 90 min. The optical density at 600 nm (OD_600_) of overnight cultures of PAO1 or *ΔkatA* was adjusted to 0.4 before 100-fold dilution in LB medium supplemented with 2% 2-hydroxyethyl-agarose (Sigma-Aldrich, Brøndby, Denmark) and 50 μl was loaded into 96-well microtiter plates (Nucleon Delta Surface; Thermo Fisher Scientific, Waltham, MA, USA) to achieve a cell loading of ≈10^6^ cells · ml^−1^. The medium was supplemented with NaNO_3_ (1 mM) (Sigma-Aldrich) to enable anaerobic respiration. The supernatant was replaced daily with 50 μl of LB medium supplemented with 1 mM NaNO_3_. Microtiter plates were covered with Parafilm (Bemis, Neenah, WI, USA) and lid and were incubated under anoxic conditions at 37°C for 3 days to establish mature biofilms. The density of mature untreated PAO1 and *ΔkatA* biofilms was 7.7 × 10^6^ CFU · ml^−1^ and 7.6 × 10^6^ CFU · ml^−1^, respectively, under anaerobic growth conditions. Treatment with ciprofloxacin was initiated by replacing the supernatant with 50 μl of a ciprofloxacin solution in LB medium (supplemented with 1 mM NO_3_^−^) in 2-fold dilutions from 0 to 2 mg · liter^−1^. The plates were then further incubated for 90 min under anoxic or HBO conditions. At the termination of experiments, the supernatant was discarded and the agarose-embedded PAO1 biofilms were placed in 2.95 ml of phosphate-buffered saline (PBS) (Substrate Department, Panum Institute, Copenhagen, Denmark) before resuspension for 15 to 20 s in a homogenizer (SilentCrusher M; Heidolph, Schwabach, Germany). Quantitative bacteriology was performed by standard microbiological methods after incubation overnight at 37°C.

### Hyperbaric oxygen treatment.

Agarose-embedded bacteria were exposed to HBOT (100% O_2_) at a pressure of 280 kPa (2.8 bar) at 37°C in a hyperbaric oxygen chamber (OXYCOM 250 ARC; Hypcom Oy, Tampere, Finland). The HBOT sequence consisted of pressurization over 5 min to a pressure of 280 kPa. The pressure was then applied for 90 min followed by 5 min of decompression. A constant temperature at 37°C in the biofilm samples was established by a circulating water system heater (FL300; Julabo, Seelbach, Germany) placed underneath the microtiter plates in the hyperbaric oxygen chamber.

### Sectioning and microscopy of agarose embedded biofilm samples.

Larger amounts of agarose-embedded biofilms were grown anaerobically with NO_3_^−^ for 3 days in 24-well microtiter plates as described above before subjection to similar treatment with ciprofloxacin and HBOT as the 96-well plate biofilm assays.

### Microscopy and image analysis.

With the use of a sterile 5-mm biopsy punch a cylindrical sample was taken from the central part of the wells in the 24-well microtiter plates. The cylindrical gel samples were cut into two halves, each with a flat cut side. The cut samples were stained by applying 100 μl of a live/dead-stain mix of Syto9 (5 μM; Molecular Probes, USA) and propidium iodide (PI) (20 μM; Thermo Fisher, USA) in MiliQ water. The stained samples were incubated in the dark for 15 min at room temperature before being placed flat-cut-side down on coverslips.

Samples were evaluated by confocal laser scanning microscopy (CLSM) on an LSM 880 Zeiss inverted microscope running Zen 2012 (Zeiss, Germany). The samples were imaged at 100× magnification by parallel tracks running 488-nm and 561-nm lasers exciting Syto9 and PI, respectively. Samples were imaged with a 1 × 6-tile scan (1,416 μm × 7,091 μm) and over a depth of 136 μm in the z-direction. Obtained z-stacks were rendered into three-dimensional (3D) projections and created in Imaris 8.3 (Bitplane, Switzerland).

Size and biomass of aggregates in CLSM image were measured with the use of Measure Pro Expansion to Imaris 8.3. An isosurface was applied over the Syto9-stained biomass as well as over biomass stained with PI. Isosurface particles larger than 100 μm^3^ were consisted. All aggregates within a depth of 100 μm from the surface of the gel were measured and returned as a measured volume. The radius of aggregates was calculated based on the assumption that aggregates were spherical. For fractionation of live and dead cells the sum of biomass between Syto9 and PI was used as total biomass. A fraction of both Syto9 and PI of the total biomass was then used as an estimate of live and dead cells.

### Oxygen measurements.

A 3-day-old untreated biofilm in a 24-well microtiter plate was treated for 90 min with HBOT. Within 1 min of ending the experiment the microtiter plate was positioned on a heated metal rack kept at 37°C and vertical microprofiles of O_2_ concentration were recorded using a computer-controlled micromanipulator (Pyro Science GmbH, Germany) equipped with a fiber-optic O_2_ microsensor (50 μm tip diameter; Pyro Science GmbH, Germany) that was connected to a fiber-optic O_2_ meter (FireSting2; Pyro Science GmbH, Germany). The microsensor was calibrated according to the manufacturer's recommendations (air saturated and O_2_-free water). As the sample was kept at 37°C, this temperature was set as the measurement temperature in the software. The microsensor was positioned manually at the base of the biofilm sample and profile measurements were taken by moving the sensor in vertical steps of 100 μm through the biofilm sample. Positioning and data acquisition were controlled by dedicated software (Profix version 4.51; Pyro Science).

### Oxygen diffusion (control).

Diffusion of oxygen in gels without cells was compared between agarose concentrations of 0.125% to 2% with an NaCl concentration of 0.9 g · liter^−1^. The gels were placed in test tubes of 65 mm height and an inner diameter of 12 mm and left to congeal. Heights of the agarose gels ranged from 21 to 41 mm. A total of 100 μl saline water (0.9 g · liter^−1^) was then added on top of the gel to avoid drying and the tubes were sealed with Parafilm. The test tubes were placed in an anaerobic chamber (Concept 400; Baker Ruskinn) at 37°C for at least 8 days to deoxygenate. The tip of the fiber-optic O_2_ micro sensor (OXR50-UHS; Pyroscience) was then positioned at 6 mm depth and the oxygen level was recorded under normoxic conditions as the gel reoxygenated.

### Statistical methods.

Statistical significance was evaluated by ordinary one- or two-way analysis of variance (ANOVA) followed by Dunnett's or Bonferroni's multiple-comparison test, respectively, and by Student's *t* test. A *P* value of ≤0.05 was considered statistically significant. Data from at least 3 independent experiments were compared. Tests were performed with GraphPad Prism 6.1 (GraphPad Software Inc., La Jolla, CA) and Microsoft Excel (Microsoft Corp., Redmond, WA).
